# Incidence and Associated Factors of Schneiderian Membrane Perforation in Maxillary Sinus Floor Elevation: A Meta-Analysis

**DOI:** 10.1016/j.identj.2026.109756

**Published:** 2026-07-23

**Authors:** Yanmei Liu, Qiuyu Zhou, Enhong Li, Xuefen Yu

**Affiliations:** a Stomatology Hospital, School of Stomatology, Zhejiang University School of Medicine, Zhejiang Provincial Clinical Research Center for Oral Diseases, Key Laboratory of Oral Biomedical Research of Zhejiang Province, Cancer Center of Zhejiang University, Hangzhou, China; b School of Medicine, Zhejiang University, Hangzhou, China; c Central Laboratory, The First Hospital of Hebei Medical University, Shijiazhuang, China

**Keywords:** Maxillary sinus floor elevation, Schneiderian membrane perforation, Sinus septa, Smoking, meta-analysis

## Abstract

**Introduction and aims:**

Schneiderian membrane perforation (SMP) is a common intraoperative event during maxillary sinus floor elevation (MSFE), yet contemporary estimates of its incidence and determinants remain inconsistent, leaving preoperative assessment, surgical planning, and perioperative counselling insufficiently informed. This systematic review and meta-analysis synthesized evidence on SMP incidence and prespecified anatomical, patient-related, and procedural factors associated with intraoperative perforation.

**Methods:**

Six databases were searched from inception to October 2025 for observational studies reporting intraoperative SMP incidence and/or associated factors during MSFE. Two reviewers independently performed study selection, data extraction, and quality appraisal (AHRQ for cross-sectional studies; Newcastle–Ottawa Scale for cohort studies). Random-effects meta-analyses pooled incidence (patient- and surgery-level) and odds ratios (ORs).

**Results:**

Twenty-six studies (4519 patients) were included. The pooled SMP incidence was 0.19 (95% confidence interval [CI] 0.14-0.24; *I*² = 91.4%) per patient and 0.19 (95% CI 0.15-0.23; *I*² = 91.9%) per surgery. Sinus septa (OR = 3.56, 95% CI = 1.82-6.96) and smoking (OR = 2.57, 95% CI = 1.47-4.47) were associated with higher odds of perforation, whereas other prespecified factors showed no significant pooled effects. Findings were robust across sensitivity analyses, and no marked small-study effects were detected for the main pooled outcomes.

**Conclusions:**

SMP occurs in approximately one in five MSFE procedures. Preoperative identification of septa and targeted counselling for smokers may improve preoperative assessment and contingency planning. Future studies should adopt standardized CBCT measurement landmarks and report perforation phenotype and management.

**Clinical relevance:**

Findings support structured preoperative assessment and informed consent focused on septa and smoking.

## Introduction

Maxillary sinus floor elevation (MSFE) is a foundational surgical procedure in implant dentistry aimed at overcoming insufficient bone volume in the atrophic posterior maxilla, a common consequence of tooth loss, physiological bone resorption, and sinus pneumatization.^1^ By elevating the Schneiderian membrane (SM) to create a contained space for bone graft material, MSFE increases subantral bone height and facilitates stable implant placement and subsequent osseointegration.^2^ This technique is generally regarded as a predictable option for the functional rehabilitation of the posterior maxilla and broadens treatment options for patients who would otherwise be ineligible for conventional implant therapy.^3^

Despite its high success rate, MSFE is associated with several complications, among which SM perforation (SMP) is the most frequently encountered.^4^ The reported incidence of SMP varies widely across the literature, ranging from 7% to 58%.^5^ Membrane perforation has been associated with graft material migration, postoperative infection, sinusitis, and impaired new bone formation, potentially compromising the success of both the augmentation procedure and subsequent implant placement.^6^^,^^7^ A meta-analysis involving 2947 patients reported an association between intraoperative SMP during MSFE and a higher likelihood of implant failure.^8^ Therefore, careful handling and preservation of SM integrity are critical to achieving predictable, successful outcomes in MSFE procedures.

The development of SMP can be explained by both intrinsic biomechanical characteristics of the SM and extrinsic anatomical and procedural factors encountered during surgery. Experimental data indicate that perforation occurs when local tension exceeds the membrane’s elastic capacity.^9^ In clinical practice, such tension is shaped by patient-specific anatomy and operative manipulation during membrane elevation.^10^ Among the investigated anatomical variables, sinus septa have been associated with higher odds of perforation during MSFE.^11^ Other anatomical factors, such as residual ridge height (RRH) and SM thickness (SMT), as well as nonanatomical factors including surgical approach, smoking, and age, have also been investigated as potential contributors to SMP.^12^, ^13^, ^14^, ^15^, ^16^ Previous meta-analyses have examined intraoperative SMP incidence and selected associated factors, but most focused on isolated variables and reached inconsistent conclusions.^12^^,^^17^ An updated and comprehensive synthesis is therefore needed. The present study aimed to (1) estimate the current overall incidence of SMP during MSFE and (2) assess the association between SMP and key anatomical and nonanatomical associated factors, thereby informing evidence-based preoperative assessment and surgical decision-making.

## Materials and methods

### Protocol and registration

The design and reporting of this systematic review and meta-analysis followed the Preferred Reporting Items for Systematic Reviews and Meta-Analyses (PRISMA) 2020 statement (see Supplementary Table S1 for the PRISMA checklist).^18^ The review protocol was prospectively registered on the International Prospective Register of Systematic Reviews (PROSPERO) in September 2025 (CRD420251273886).

### Information sources and search strategy

A comprehensive literature search was conducted across six electronic databases (Web of Science, PubMed, Scopus, Embase, CINAHL, and the Cochrane Library) from database inception to October 2025. Controlled vocabulary terms (eg, MeSH in PubMed and Emtree in Embase) and free-text keywords were combined for two concepts: MSFE and membrane perforation. Full search strategies for each database are provided in Supplementary Table S2. No language restrictions were applied. We did not actively search grey literature; this decision was made to prioritize peer-reviewed published data with established quality control, which is the standard approach in most systematic reviews of surgical complications. However, the reference lists of included studies were hand-searched to identify additional eligible reports.

### Eligibility criteria

The research question was formulated using the Population, Exposure, Comparison, Outcomes, and Study design (PECOS) framework: ‘In patients undergoing MSFE (P), are anatomic, patient-related, or procedure-related exposures (E), compared with their absence or reference categories (C), associated with intraoperative SMP (O) in observational studies (S)?’

Studies were included if they met all of the following criteria: (1) enrolled patients undergoing MSFE, (2) reported the intraoperative incidence of SMP and/or extractable data on prespecified associated factors, and (3) adopted an observational design (cohort, case-control, or cross-sectional). Because the review was designed to evaluate observational associations rather than intervention effects, randomized controlled trials and other interventional studies were outside the prespecified scope and were therefore not eligible. A meta-analysis for a given associated factor was performed only when data were available from at least three studies.

Studies were excluded if they were in vitro or animal studies, reviews/editorials, case reports, conference abstracts, or if data were incomplete or the full text was unavailable.

### Study selection and data extraction

Two reviewers (Y.M.L. and Q.Y.Z.) independently performed the study selection and data extraction. First, all retrieved records were imported into EndNote X9 to remove duplicates. Subsequently, titles and abstracts were screened for preliminary eligibility, followed by full-text screening for final inclusion. Data were extracted using a predesigned, standardized form. The following information was extracted from each included study: first author, publication year, country, sample size, study design, participant characteristics, incidence of membrane perforation, and anatomical or surgical associated factors. All decisions were cross-checked between the two reviewers. Any discrepancies were resolved through discussion or, if necessary, by consultation with a third reviewer (E.H.L.).

### Quality appraisal

Methodological quality was assessed independently by two reviewers (Y.M.L. and Q.Y.Z.). Cross-sectional studies were assessed using the 11-item checklist recommended by the Agency for Healthcare Research and Quality (AHRQ), with total scores interpreted as follows: 0 to 3 (low quality), 4 to 7 (moderate quality), and 8 to 11 (high quality).^19^ Cohort and case-control studies were evaluated using the Newcastle–Ottawa Scale (NOS), which rates studies across three domains: selection, comparability, and outcome. The total NOS score ranges from 0 to 9; studies with scores ≥7 were considered high quality, scores of 4 to 6 indicated moderate quality, and scores of 0 to 3 indicated low quality.^20^ Disagreements in quality ratings were resolved through consensus or by adjudication from the third reviewer (E.H.L.).

### Data synthesis and statistical analysis

Given the anticipated heterogeneity across studies, data were harmonized before quantitative synthesis. Incidence estimates were analysed separately at the patient and surgery levels, and each study contributed only one effect estimate to each meta-analysis of an associated factor. When definitions were incompatible or fewer than three studies were available, findings were summarized narratively rather than pooled.

(1) Incidence pooling

The incidence of SMP was pooled as proportions using random-effects models and synthesized separately at the patient and surgery levels. To stabilize variances, proportions were transformed using the Freeman–Tukey double-arcsine method and then back-transformed for presentation. Random-effects models were applied to account for anticipated clinical and methodological heterogeneity across studies.

(2) Associated factor pooling

For prespecified associated factors, dichotomous predictors were synthesized as odds ratios (ORs) with 95% confidence intervals (CIs). Adjusted ORs were extracted preferentially when available; otherwise, crude estimates were derived from raw data. When a study reported data at both the patient and surgery levels for the same predictor, we prioritized patient-level data to minimize unit-of-analysis errors; surgery-level data were used only when patient-level data were unavailable.

For predictors reported using multiple cut-offs within the same study (eg, SMT), we extracted the estimate based on the prespecified threshold when available; otherwise, we selected the cut-off closest to the prespecified threshold or the primary definition stated by the authors, ensuring that each study contributed only one effect size per meta-analysis.

If a study reported multiple mutually exclusive strata (eg, by surgical approach or anatomical site), we derived a single study-level estimate by combining strata within the study. This was achieved by aggregating raw 2 × 2 data when available or by inverse-variance pooling on the log scale. When statistical combination was not feasible, the estimate from the most clinically representative stratum was extracted, and the decision was documented. ORs were pooled on the log scale using random-effects models. Continuous predictors were synthesized as mean differences with 95% CIs. Random-effects models were used throughout to account for anticipated clinical and methodological heterogeneity.

(3) Heterogeneity and publication bias

Between-study heterogeneity was assessed using Cochran’s *Q* and quantified with the *I*² statistic. Prespecified subgroup analyses were conducted according to surgical approach (transcrestal vs lateral window) and timing of implant placement (partial simultaneous, fully simultaneous, and staged). Subgroup-specific pooled estimates and within-subgroup heterogeneity were calculated using random-effects models, and between-subgroup differences were evaluated using a test for subgroup differences (Cochran’s *Q* between subgroups). Sensitivity analyses included leave-one-out analyses and, when applicable, the exclusion of low-quality studies. Publication bias was assessed through visual inspection of funnel plots and formally tested using Egger’s regression and Begg’s rank correlation tests when at least 10 studies were available. All analyses were performed in Stata 17.0 (StataCorp) using two-sided tests, with *P* < .05 considered statistically significant.

### Certainty of evidence

The certainty of evidence for each outcome was assessed using the Grading of Recommendations Assessment, Development, and Evaluation (GRADE) framework.^21^^,^^22^ Two reviewers (Y.M.L. and Q.Y.Z.) independently rated evidence across five domains: risk of bias, inconsistency, indirectness, imprecision, and publication bias. Final certainty was categorized into four levels: high, moderate, low, or very low.^23^

## Results

### Study selection and characteristics

**Fig. 1:**
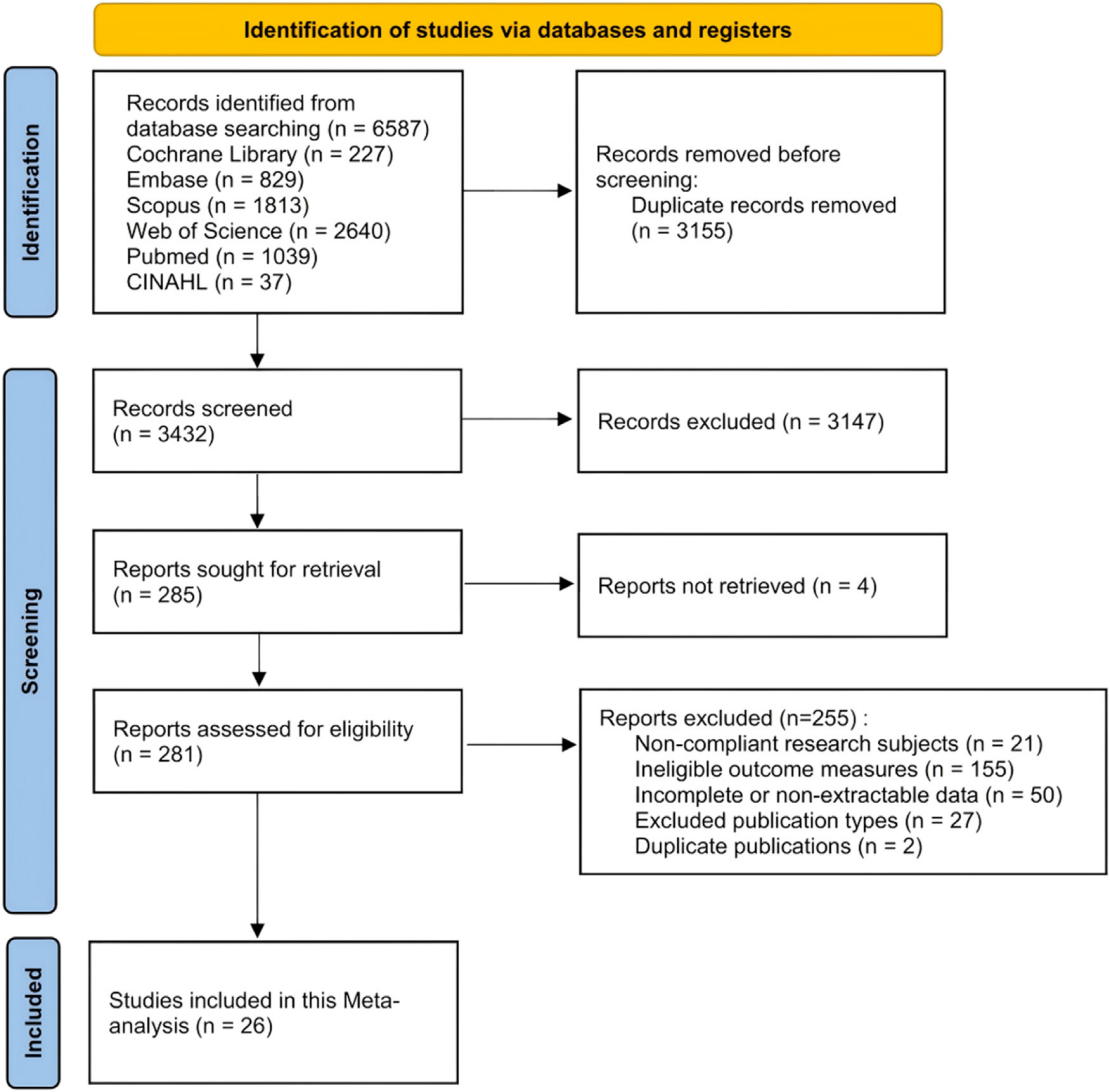
PRISMA flow diagram of study selection.

Figure 1 summarizes the study selection process. In total, 26 studies^5^^,^^16^^,^^24^, ^25^, ^26^, ^27^, ^28^, ^29^, ^30^, ^31^, ^32^, ^33^, ^34^, ^35^, ^36^, ^37^, ^38^, ^39^, ^40^, ^41^, ^42^, ^43^, ^44^, ^45^, ^46^, ^47^ published between 2003 and 2024 met the eligibility criteria, comprising 4519 patients. Twenty-four studies reported the number of MSFE surgeries, yielding 5101 surgeries in total; the remaining 2 did not provide surgery counts. Across the included studies, 3029 implants were placed. The studies were conducted in 20 countries or regions across Asia, Europe, North America, and South America, including 5 multicentre investigations. Key study characteristics are summarized in Table 1.

**Table 1:** General characteristics of included studies (*n* = 26).

Study	Y	Country	Study type	Sample size	Surgical volume	Surgical approach	Risk factors	Quality assessment tool	Score (NOS/AHRQ)	Quality level
Li^24^	2024	China	Cohort study	87	117	Transcrestal approach	B, L	NOS	8	High
Mazor^5^	2024	Israel, Portugal, Brazil, Croatia, India, USA	Cross-sectional study	621	670	Transcrestal approach	G	AHRQ	4	Moderate
Gao^25^	2023	China	Cohort study	259	340	Transcrestal approach	F, I, J, K, L	NOS	9	High
Prajapati^26^	2023	USA	Cohort study	99	122	Lateral window approach	F, I, K, L	NOS	7	High
Nemati^27^	2023	Iran, Spain	Cohort study	140	140	Lateral window approach	A, B, C, D, E, F, H, K, L	NOS	8	High
Basma^28^	2021	USA	Cross-sectional study	209	251	Lateral window approach	A, C, E	AHRQ	6	Moderate
Boyacıgil^29^	2021	Turkey	Cohort study	25	44	Transcrestal approach	B, C	NOS	8	High
Pizzini^30^	2021	USA	Cohort study	166	202	Lateral window approach	A	NOS	8	High
Zhou^31^	2021	China	Cross-sectional study	306	320	Lateral window approach	B, K	AHRQ	5	Moderate
Shao^16^	2021	China	Cross-sectional study	278	278	Lateral window approach	F, L	AHRQ	6	Moderate
Krennmair^32^	2020	Germany, Austria	Cohort study	355	434	Lateral window approach	B, C, D, E, F, H, I, K, L	NOS	9	High
Park^33^	2019	Korea	Cross-sectional study	63	65	Lateral window approach	B, C, D, I, K, L	AHRQ	4	Moderate
Marin^34^	2019	Bosnia and Herzegovina, Austria	Cross-sectional study	121	137	Lateral window approach	E, F, G, H, J, K	AHRQ	4	Moderate
Tükel^35^	2018	Turkey	Cross-sectional study	120	120	Lateral window approach	F, K, L	AHRQ	5	Moderate
Lum^36^	2017	USA	Cross-sectional study	167	/	Lateral window approach	B, C, D, K, L	AHRQ	6	Moderate
Irinakis^37^	2017	Canada	Cohort study	67	79	Lateral window approach	F, J, K, L	NOS	8	High
Monje^38^	2016	Spain	Cohort study	40	/	Lateral window approach	A	NOS	8	High
Lin^39^	2016	China	Cohort study	73	81	Lateral window approach	B	NOS	8	High
Merli^40^	2016	Italy	Cross-sectional study	124	148	Lateral window approach	/	AHRQ	6	Moderate
Rapani^41^	2016	Italy	Cross-sectional study	200	200	Lateral window (*n* =100)/transcrestal (*n* = 100)	/	AHRQ	5	Moderate
Wen^42^	2015	China	Cohort study	122	185	Transcrestal approach	B	NOS	8	High
Schwarz^43^	2015	Austria	Cross-sectional study	300	407	Lateral window approach	F, K, L	AHRQ	5	Moderate
Thomas^44^	2014	Switzerland, Chile, Denmark	Cross-sectional study	77	77	Lateral window approach	A, B, C, E, F, G, H, J, K, L	AHRQ	7	Moderate
Yilmaz^45^	2012	Turkey	Cross-sectional study	44	64	Lateral window approach	/	AHRQ	5	Moderate
Hernández^46^	2008	Spain	Cross-sectional study	338	474	Lateral window approach	/	AHRQ	4	Moderate
Kasabah^47^	2003	Czech Republic	Cross-sectional study	118	146	Lateral window approach	F, L	AHRQ	4	Moderate

‘A’ indicates ‘lateral wall bone thickness’, ‘B’ indicates ‘membrane thickness’, ‘C’ indicates ‘residual bone height’, ‘D’ indicates ‘age’, ‘E’ indicates ‘edentulous area’, ‘F’ indicates ‘sinus septa’, ‘G’ indicates ‘operation site’, ‘H’ indicates ‘operation side’, ‘I’ indicates ‘diabetes’, ‘J’ indicates ‘simultaneous vs staged implant placement’, ‘K’ indicates ‘sex’, ‘L’ indicates ‘smoking’. The quality score represents the Newcastle–Ottawa Scale (NOS; 0-9) for cohort studies and the Agency for Healthcare Research and Quality checklist (AHRQ; 0-11) for cross-sectional studies; the two tools are not directly comparable. Quality level was categorized as high/moderate/low based on NOS scores of 7 to 9/4 to 6/0 to 3 and AHRQ scores of 8 to 11/4 to 7/0 to 3.

AHRQ, Agency for Healthcare Research and Quality; NOS, Newcastle–Ottawa Scale.

15 studies used cross-sectional designs, and 11 used cohort designs. Most studies enrolled adult patients with partial or complete edentulism in the posterior maxillary region undergoing MSFE. Several studies further restricted eligibility according to RRH, smoking status, or systemic conditions.

The lateral window approach was reported in 21 studies, and the transcrestal approach in 6 studies, including 1 study that evaluated both approaches. In some reports, the surgical approach varied by case selection or time period within the same centre, and implant placement was performed either simultaneously or in a staged manner.

Evaluated variables spanned anatomical, patient-related, and procedure-related domains. The most frequently assessed variables were sinus septa, SMT, smoking status, and sex, while definitions and measurement protocols for several exposures varied across studies.

### Risk of bias

Cross-sectional studies were appraised using the AHRQ checklist.^19^ Scores ranged from 4 to 7 (median, 5), indicating overall moderate quality. The primary sources of bias were incomplete reporting of sampling frames and consecutive enrolment, insufficient description of exposure and outcome assessment, and limited control of potential confounders.

Cohort studies were assessed using the NOS.^20^ The NOS score ranged from 7 to 9 (median, 8), suggesting generally good methodological quality, with high ratings for participant selection and outcome domains. However, systematic adjustment for confounders remained suboptimal in some studies. Overall, 11 studies were rated as high quality and 15 as moderate quality; no study met low-quality criteria (Table 1).

### Descriptive findings

Substantial clinical and methodological heterogeneity was observed across studies in the definitions and measurement protocols of exposures and outcomes. SMP was generally recorded as a binary intraoperative event, whereas detailed characterization of perforation size or management was rarely reported. Considerable variation was also noted in anatomical assessments, particularly for SMT and lateral wall thickness, which were measured using different CBCT landmarks and categorised with variable cut-offs. Definitions of patient- and procedure-related variables, such as smoking and simultaneous vs staged implant placement, also differed across studies. These inconsistencies likely contributed to the heterogeneity observed in the pooled analyses and were considered when interpreting the results. Therefore, quantitative pooling was restricted to outcomes and associated factors with sufficiently comparable definitions and extractable data. Variables with incompatible definitions or insufficient contributing studies were not pooled and were considered narratively.

### Meta-analysis of perforation incidence

**Fig. 2:**
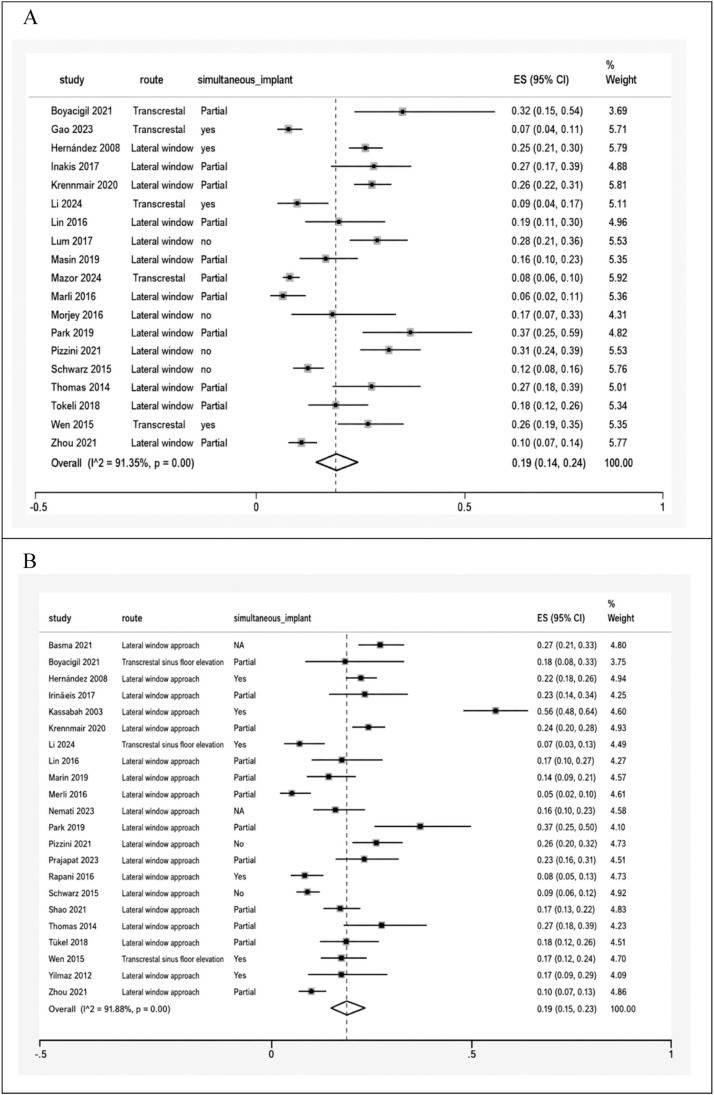
Forest plots of Schneiderian membrane perforation incidence. (A) Pooled incidence at the patient level (19 studies). (B) Pooled incidence at the surgery level (22 studies).

Nineteen studies reported SMP incidence at the patient level. Under a random-effects model, the pooled incidence of SMP was 0.19 (95% CI 0.14-0.24) at the patient level (*I*² = 91.4%, *P* < .001; Figure 2A). At the surgery level, 22 studies contributed data (5101 surgeries), yielding a pooled incidence of 0.19 (95% CI 0.15-0.23) (*I*² = 91.9%, *P* < .001; Figure 2B). This finding suggests that approximately one in five patients undergoing MSFE experience membrane perforation.

### Meta-analysis of associated factors

A total of 11 prespecified associated factors were synthesized using random-effects models. Depending on the factor, the number of included studies ranged from 4 to 13, and the statistical unit (patient or surgery) varied across primary studies.

**Fig. 3:**
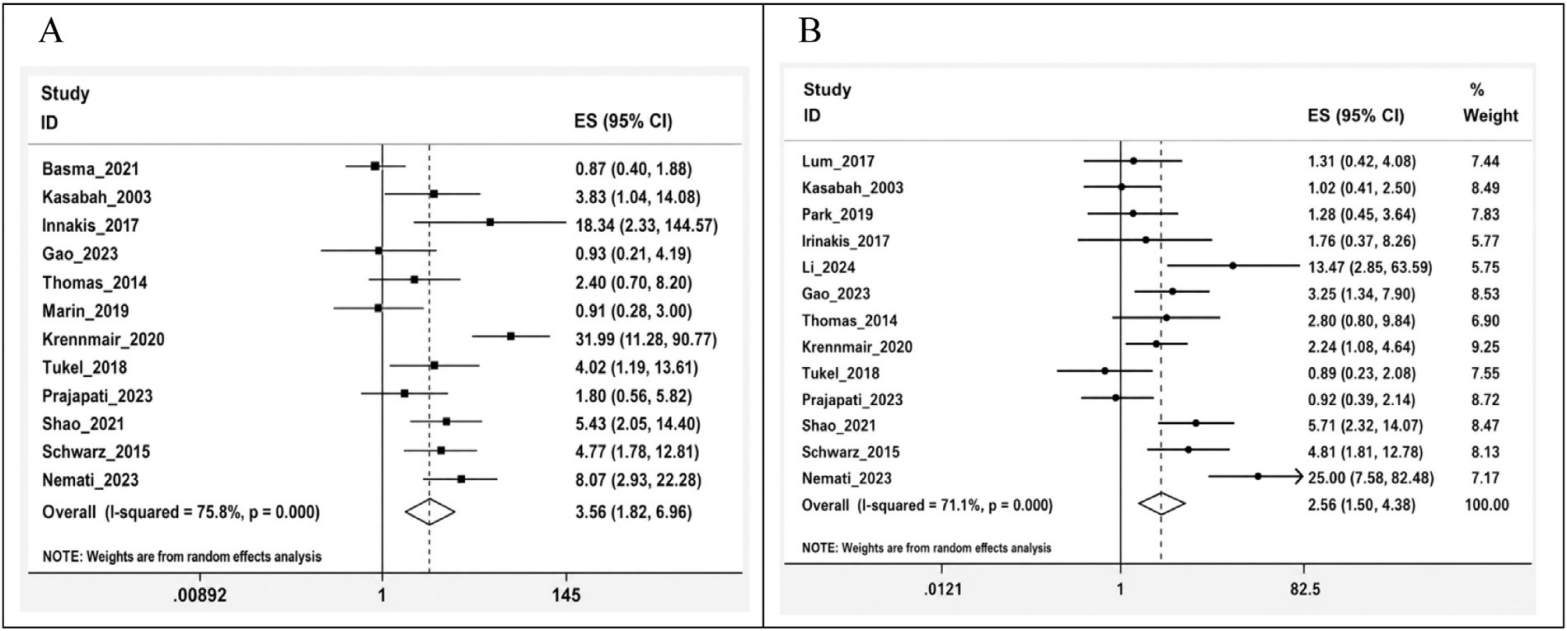
Forest plots of the two main associated factors for Schneiderian membrane perforation. (A) Sinus septa. (B) Smoking.

Sinus septa showed the most consistent association with SMP. Twelve studies yielded a pooled OR of 3.56 (95% CI 1.82-6.96; *I*² = 76.0%), indicating higher odds of perforation when septa were present (Figure 3A).

Neither SMT nor lateral wall thickness showed a statistically significant pooled association with SMP, whether analysed as continuous variables or dichotomized according to prespecified thresholds. Heterogeneity of these analyses was high. Variation in CBCT landmarks, imaging protocols, and cut-offs for ‘thick’ membranes likely contributed to between-study variability (Supplementary Figure S1A-C).

RRH was further analysed as a supplementary continuous anatomical variable. The pooled estimate showed lower RRH in perforation cases, although substantial heterogeneity was observed (SMD = −0.46, 95% CI −0.80 to −0.12; *I*² = 80.3%; Supplementary Figure S1D).

Smoking was associated with higher odds of perforation (13 studies; pooled OR 2.57; 95% CI 1.47-4.47; *I*² = 71.7%; Figure 3B). Pooled effects for sex, age, and diabetes mellitus were not statistically significant and were accompanied by moderate to high heterogeneity (Supplementary Figure S1E-G).

Simultaneous vs staged implant placement did not show a statistically significant pooled association with SMP (4 studies; pooled OR 1.87; 95% CI 0.99-3.52; Supplementary Figure S1H). Other evaluated factors, including surgical side and edentulous region/type, also showed no statistically significant pooled associations.

### Subgroup analyses

In subgroup analyses at the patient level by surgical approach, the pooled incidence was 0.14 (95% CI 0.07-0.22) for the transcrestal approach and 0.20 (95% CI 0.16-0.26) for the lateral window approach, with no evidence of between-subgroup difference (*P* = .158). At the surgery level, pooled incidences were 0.13 (95% CI 0.06-0.22) and 0.20 (95% CI 0.15-0.25) for the transcrestal and lateral window approaches, respectively (*P* = .221; Table 2; Supplementary Figure S2).

**Table 2:** Subgroup analysis of sinus membrane perforation incidence.

Factor	Statistical unit	Subgroup	Pooled incidence	95% CI	*I*² within subgroup (%)	*P* for heterogeneity within subgroup (Cochran’s *Q*)	*P* for subgroup differences (Cochran’s *Q* between subgroups)
Surgical approach	Patient	Transcrestal	0.14	0.07-0.22	89.38	*P* < .001	0.158
	Patient	Lateral window	0.20	0.16-0.26	88.01	*P* < .001	
	Surgery	Transcrestal	0.13	0.06-0.22	88.00	*P* < .001	0.221
	Surgery	Lateral window	0.20	0.15-0.25	92.64	*P* < .001	
Timing of implant placement	Patient	Partial simultaneous	0.19	0.13-0.26	91.27	*P* < .001	0.765
	Patient	Fully simultaneous (yes)	0.16	0.07-0.28	93.93	*P* < .001	
	Patient	Staged only (no)	0.22	0.12-0.34	90.97	*P* < .001	
	Surgery	Staged only (no)	0.18	0.10-0.30	93.95	*P* < .001	0.984
	Surgery	Partial simultaneous	0.18	0.14-0.23	85.27	*P* < .001	
	Surgery	Fully simultaneous (yes)	0.20	0.09-0.33	96.14	*P* < .001	
Overall (all studies)	Patient	—	0.19	0.14-0.24	91.35	*P* < .001	—
	Surgery	—	0.19	0.15-0.23	91.88	*P* < .001	—

Patient-level incidence used the number of patients as the denominator; surgery-level incidence used the number of sinus floor elevation procedures as the denominator.

When stratified by timing of implant placement, patient-level pooled incidences for ‘partial simultaneous’, ‘fully simultaneous’, and ‘staged only’ strategies were 0.19 (95% CI 0.13-0.26), 0.16 (95% CI 0.07-0.28), and 0.22 (95% CI 0.12-0.34), respectively (*P* = .765 between subgroups). Surgery-level analyses yielded similar findings (*P* = .984 between subgroups; Table 2; Supplementary Figure S2).

### Sensitivity analyses

Leave-one-out sensitivity analyses showed that pooled incidence estimates at both the patient and surgery levels were robust to omission of individual studies. For the key associated factors (sinus septa and smoking), pooled ORs remained stable with no change in statistical significance. Because no study was rated low quality, sensitivity analyses focused on leave-one-out methods. For analyses with high heterogeneity (eg, membrane thickness and lateral wall thickness), excluding studies that contributed most to heterogeneity reduced *I*² somewhat but did not materially change conclusions.

### Publication bias

**Fig. 4:**
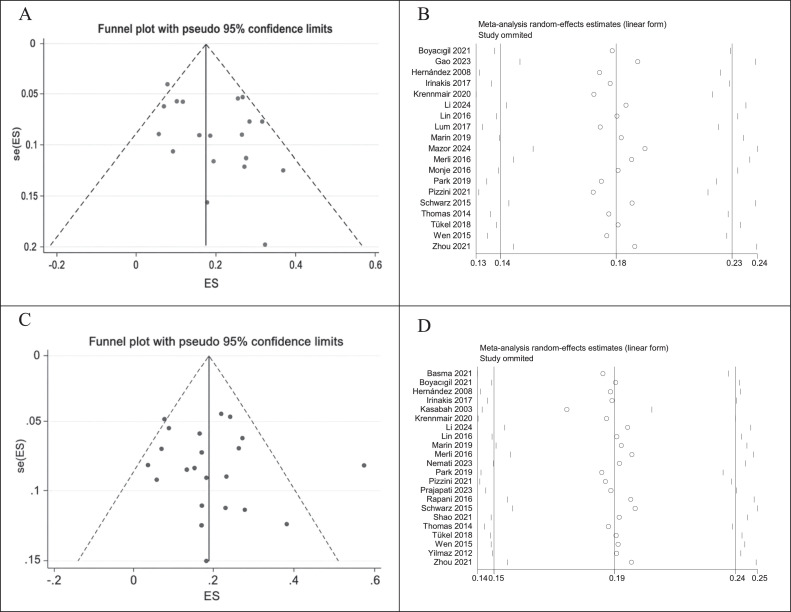
Funnel plots and Egger’s regression plots for Schneiderian membrane perforation incidence. (A and B) Patient level. (C and D) Surgery level.

For the pooled analyses of SMP incidence at both the patient and surgery levels, funnel plots were broadly symmetrical, and Begg’s and Egger’s tests (available because ≥10 studies contributed) yielded *P* values >.05 (Figure 4). For the two main associated factors (sinus septa and smoking), funnel plots similarly showed no marked asymmetry, and Egger’s regression did not indicate significant small-study effects (Supplementary Figure S3). For other associated factors with fewer than 10 contributing studies, we restricted the assessment to a qualitative inspection without formal statistical tests.

### GRADE assessment

GRADE assessment revealed that the certainty of evidence ranged from very low to low across all outcomes. The pooled SMP incidence was rated as very low, downgraded for very serious inconsistency (*I*² > 90%) and serious risk of bias. Other very low outcomes (membrane thickness, lateral wall thickness, age, edentulous region) were downgraded for high risk of bias, serious inconsistency, or imprecision. All remaining outcomes were rated low. As all included studies were observational, causal inference is limited. Given the low to very low certainty of the evidence, the results should be interpreted with caution. Detailed information is presented in Supplementary Table S3.

## Discussion

### Incidence

This meta-analysis synthesized 26 studies published between 2003 and 2024 to estimate the incidence of SMP during MSFE and to examine factors associated with intraoperative perforation. The evidence base included 4519 patients from 20 countries or regions, including five multicentre studies. The pooled SMP incidence was approximately 19%, whether calculated per patient or per procedure, although heterogeneity was substantial (*I*² > 90% in the primary analyses). This estimate is broadly consistent with previous syntheses and supports the need to anticipate SMP during planning and to address it explicitly in informed consent.^48^ The heterogeneity likely reflects both genuine clinical variability and differences in methods and reporting, including SMP definitions (any visible tear vs defects requiring repair), the unit of analysis (patient vs procedure), and variation in approach selection and case complexity. Unlike previous reviews that focused on isolated variables, the present study synthesized incidence estimates together with multiple anatomical, patient-related, and procedural-associated factors within a single framework.

Subgroup analyses showed numerically lower pooled incidence estimates for the transcrestal approach than for the lateral window approach, although no significant between-subgroup difference was detected. The result is clinically plausible because lateral access typically requires a larger osteotomy and more extensive membrane elevation. However, these comparisons should be interpreted cautiously because approach selection is primarily driven by baseline anatomy, particularly residual bone height, sinus contour, and the required elevation distance. This introduces confounding by indication, so the chosen approach may be a marker of case complexity rather than an independent determinant of SMP.^49^

Leave-one-out sensitivity analyses and assessments of small-study effects did not materially change the pooled estimates, supporting the robustness of the incidence finding despite substantial heterogeneity.^50^

### Key associated factors

This review evaluated 11 factors potentially associated with SMP. No single variable appeared to dominate. Rather, the occurrence of perforation appears to reflect the combined effects of unfavourable anatomy, membrane susceptibility, and procedural complexity.^48^^,^^51^ Because all included studies were observational, the pooled estimates should be interpreted as associations rather than evidence of causal effects.

Among anatomical factors, sinus septa showed the most consistent association with SMP. The pooled estimate indicated approximately 3.5-fold higher odds of perforation when septa were present. Septa may disrupt the elevation plane, constrain instrument angulation, and concentrate traction during membrane elevation.^32^ Because septal morphology was inconsistently reported, some residual heterogeneity is expected. Clinically, these findings suggest the value of identifying septa preoperatively and accounting for them during surgical planning. When available, septal location, height, and orientation may be more informative than a simple present/absent classification.

**Fig. 5:**
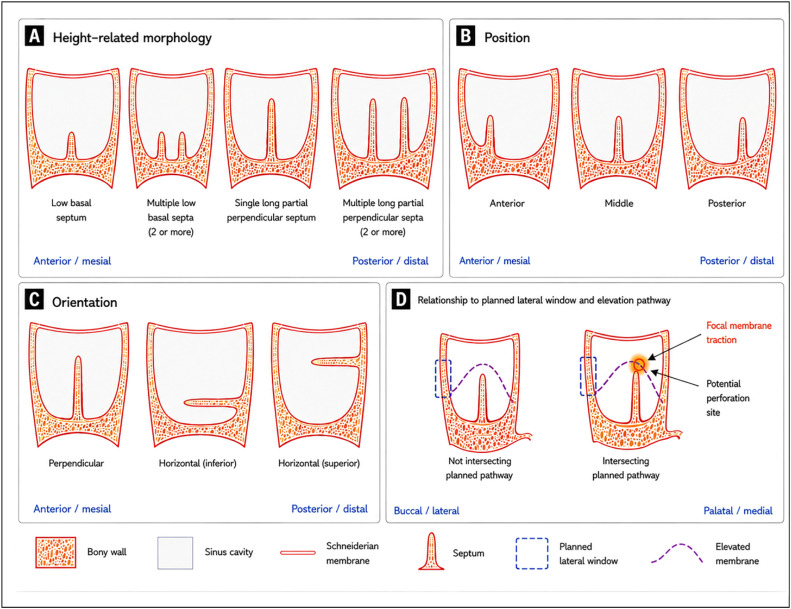
Schematic illustration of maxillary sinus septal morphology relevant to Schneiderian membrane perforation during maxillary sinus floor elevation. Notes: Panels (A-C) show simplified anterior–posterior/mesial–distal schematics of septal height-related morphology, position, and orientation. Panel (D) shows a simplified buccal–palatal cross-section illustrating the relationship between septa, the planned lateral window, and the membrane elevation pathway. The Schneiderian membrane is shown as a thin lining along the sinus wall and septal surface. When the planned elevation pathway intersects a septum, focal membrane traction may occur near the septal surface, indicating a potential site of perforation. This figure is a conceptual schematic rather than a quantitative risk scoring system.

Although the present meta-analysis mainly evaluated sinus septa as a dichotomous variable, the clinical relevance of septa is unlikely to be determined by their presence alone. Previous CBCT-based studies have shown that maxillary sinus septa vary substantially in height, number, anterior–posterior position, orientation, and configuration, and several classification systems have attempted to relate these morphological patterns to the technical difficulty of sinus lift procedures or the predicted risk of SMP.^52^ Low basal septa may have limited influence on membrane elevation, whereas long partial perpendicular septa, multiple septa, or inferiorly located horizontal septa may interfere with the elevation plane and create local points of membrane fixation. During membrane elevation, these anatomical configurations may constrain instrument angulation and concentrate traction near the septal surface, particularly when the septum intersects the planned lateral window or the membrane elevation pathway.^53^ Therefore, preoperative CBCT assessment should not only document the presence or absence of septa but also describe clinically relevant morphological features, including height-related morphology, anterior–posterior location, orientation, and spatial relationship with the planned surgical pathway.^54^ This is also consistent with the broader concept that digital and CBCT-based planning can help integrate anatomical information into surgical decision-making in implant-related procedures.^55^ A schematic illustration is provided in Figure 5.

Smoking was the host-related factor most consistently associated with higher odds of perforation (pooled OR = 2.57). Because most included studies were observational, smoking should be interpreted as a pragmatic risk marker rather than proof of causality. Nevertheless, smoking status is readily obtainable and may be incorporated into preoperative counselling, particularly in anatomically complex cases.^15^ Several biological hypotheses have been proposed, including smoking-related mucosal and microvascular alterations; however, causal mechanisms remain uncertain.^17^ Smoking may therefore be considered in perioperative risk communication.

SMT requires cautious interpretation. Although thinner membranes may be more frequent in perforation cases, findings were sensitive to CBCT measurement protocols and categorization methods. Fixed cut-offs did not show stable threshold effects. Conversely, increased SMT may reflect mucosal oedema or chronic sinus inflammation, which may be more relevant to postoperative sinus symptoms than to intraoperative tearing itself. SMT may therefore influence perforation risk differently depending on the anatomical and surgical context, rather than acting as an isolated predictor, particularly because its apparent effect is influenced by septa, sinus contour, and elevation demand.^12^^,^^46^

Lateral wall thickness did not show a stable pooled association and was characterized by substantial heterogeneity. This probably reflects underreporting of technical modifiers between wall thickness and membrane trauma, such as osteotomy method, window design, instrumentation, and operator experience.^51^ Residual bone height remains central to clinical planning. The supplementary pooled analysis suggested lower RRH in perforation cases, although the substantial heterogeneity indicates that interpretation should remain cautious. Its independent effect is difficult to isolate because residual bone height strongly influences approach selection, elevation distance, and implantation timing.^49^ For several other prespecified factors, including sex, age, diabetes mellitus, simultaneous vs staged implant placement, surgical side, and edentulous region/type, pooled effects were not statistically significant. These nonsignificant pooled findings should be interpreted cautiously, given variability in definitions, limited stratification, and likely confounding by case selection.^56^

### Heterogeneity and interpretation

Substantial heterogeneity was observed across several pooled outcomes and likely reflects both genuine clinical variability and differences in study design, data structure, and reporting.^43^ Clinically, the included studies differed in surgical approach, residual bone height, sinus anatomy, septal morphology, elevation demand, timing of implant placement, and operator-related technical factors. Methodologically, heterogeneity arose from differences in the unit of analysis, definitions of SMP, CBCT measurement landmarks, exposure cut-offs, and whether crude or adjusted estimates were reported. To improve comparability, incidence estimates were analysed separately at the patient and surgery levels, random-effects models were used throughout, adjusted estimates were prioritized when available, and multiple cut-offs or strata were harmonized into a single study-level estimate where appropriate. Therefore, the pooled estimates are better interpreted as general patterns of association across different clinical settings rather than exact estimates for individual patients.

A central interpretive issue is confounding by indication. Lower residual bone height usually indicates greater elevation demand and more frequent selection of lateral access, which limits causal interpretation of approach comparisons.^49^ In this context, the surgical approach may partly reflect baseline anatomical complexity rather than function as an independent determinant of SMP. In addition, key technical details were often poorly reported, including instrumentation, osteotomy design, window configuration, and membrane elevation techniques, which further reduces comparability across studies.

High heterogeneity does not necessarily invalidate the overall direction of the pooled findings, but it reduces the precision and generalizability of the summary estimates. For incidence outcomes, heterogeneity likely reflects differences in case complexity, surgical protocols, and reporting thresholds for perforation. For associated factors, especially SMT and lateral wall thickness, heterogeneity was further amplified by inconsistent CBCT landmarks and categorization thresholds. Accordingly, statistically significant findings such as sinus septa and smoking should be regarded as consistent associations across studies or clinically relevant associations, whereas nonsignificant findings should not be interpreted as definitive evidence of no association.

Some evidence syntheses suggest that piezoelectric devices may be associated with lower perforation rates than rotary instruments in lateral window augmentation. However, effect sizes vary across reviews and study designs, and may also reflect case selection and operator preference.^57^, ^58^, ^59^ Leave-one-out analyses indicated that the pooled incidence and the main association signals were not driven by any single study, supporting the stability of the core conclusions despite heterogeneity.^50^ Nevertheless, the GRADE assessment indicated that the certainty of evidence across all outcomes was low to very low, primarily due to the observational study designs and substantial heterogeneity. These findings should therefore be interpreted as associations rather than evidence of causality.

### Perforation outcomes and clinical implications

SMP should not be equated with surgical failure. Evidence syntheses suggest that when SMP is recognized promptly and managed appropriately intraoperatively, short- to medium-term implant survival and graft-related outcomes may be comparable to those in nonperforated cases.^60^^,^^61^ The clinical focus may therefore be better directed towards early detection and controlled management rather than treating SMP occurrence alone as a definitive prognostic indicator.

The clinical impact of SMP likely varies according to perforation phenotype. A simple present-or-absent record may be insufficient to capture this variability. Reporting perforation size, location (floor, lateral, superior, or ostium-adjacent), and repair strategy may facilitate interpretation of postoperative sinus symptoms and follow-up outcomes.^61^^,^^62^ A simple grading approach may help describe these clinical differences more clearly. Minor peripheral defects are often amenable to sealing, allowing cautious continuation of grafting. In contrast, large or unstable tears require secure containment and may justify conversion to a staged strategy, especially when close to the ostium or under high tension.

The present findings support preoperative assessment and contingency planning.^1^^,^^63^ Anatomical features such as septa, SMT, sinus morphology, residual bone height, and the planned elevation demand may be documented using standardized definitions to improve transparency and comparability. These features can inform counselling and surgical design. When multiple associated factors cluster, such as septa with smoking and limited residual bone height, teams should plan explicitly and ensure that repair materials are readily available. In parallel, routine oral hygiene instruction should be incorporated into perioperative preparation, as recent evidence supports the importance of maintaining a favourable oral environment during dental treatment.^64^ Intraoperatively, membrane handling and window design may influence the mechanical tension applied to the membrane. Avoidance of septal intersections and minimization of focal traction may reduce mechanical strain on the membrane. In anatomically demanding lateral window cases, less traumatic osteotomy or instrumentation may be considered, while recognizing that reported benefits vary across evidence syntheses.^57^^,^^59^ Postoperatively, early recognition and management of sinus symptoms remain important, particularly in patients with pre-existing mucosal thickening or other signs of compromised sinus health.

### Limitations

This review has limitations. All 26 included studies were observational (15 cross-sectional and 11 cohort studies). Protocols and reporting were heterogeneous, so pooled estimates should be interpreted as average association patterns across diverse clinical settings rather than causal or universally applicable effects. The unit of analysis also varied across studies (patient vs surgery), which may have added further variability. Definitions of SMP and its intraoperative management were not uniform. CBCT-based predictors, particularly SMT, were measured using different landmarks, imaging protocols, and cut-offs. Several comparisons were based on a limited number of studies, which reduces power for subgroup analyses. Publication bias testing is also insensitive when few studies are available. The absence of a systematic grey literature search may have introduced publication bias, as smaller or negative studies are more likely to remain unpublished; this limitation should be considered when interpreting the pooled estimates. Important technical factors were incompletely reported in many primary studies. These include surgeon experience, osteotomy design, and instrumentation choices. Such variables may partly explain heterogeneity, but could not be reliably synthesized. Future work should use prospective or well-characterized multicentre cohorts with standardized indications and surgical protocols. Studies should report perforation size, location, and repair strategy in a consistent manner and relate these details to graft behaviour, sinus complications, and implant survival.^63^ Future imaging standardization methods may improve reproducibility of anatomical measurements.^65^^,^^66^

## Conclusion

This systematic review and meta-analysis of 26 studies (4519 patients) provides an updated benchmark for SMP during MSFE, with a pooled incidence of approximately 19%. Among the evaluated factors, sinus septa and smoking emerged as the most consistent clinically relevant associated factors. Taken together, these findings may help clinicians assess anatomical and procedural complexity before surgery and plan accordingly. They also highlight priorities for future prospective research, including standardized CBCT measurements and consistent reporting of perforation phenotype and management, to further improve preoperative assessment and standardized clinical evaluation.

## Declaration of generative AI and AI-assisted technologies in the writing process

During the preparation of this work, the authors used ChatGPT in order to improve the language of the manuscript. After using this tool, the authors reviewed and edited the content as needed and take full responsibility for the content of the publication.

## Funding

This work was supported by the Zhejiang Medical and Health Science and Technology Project (grant number: 2025HY0506).

## Data availability

The data supporting the findings of this study are available from the corresponding author upon reasonable request.

## Author contributions

Yanmei Liu: Methodology, data curation, writing – original draft. Qiuyu Zhou: Data curation, formal data analysis, investigation. Enhong Li: Validation, data curation. Xuefen Yu: Methodology, writing – review and editing, supervision, funding acquisition.

## Conflict of interest

The authors declare that they have no known competing financial interests or personal relationships that could have appeared to influence the work reported in this article.

## Appendix Supplementary materials

Image, application 1

## Footnotes

Supplementary material associated with this article can be found in the online version at doi:10.1016/j.identj.2026.109756.
